# Constitutively Active FOXO1 Diminishes Activin Induction of *Fshb* Transcription in Immortalized Gonadotropes

**DOI:** 10.1371/journal.pone.0113839

**Published:** 2014-11-25

**Authors:** Chung Hyun Park, Danalea V. Skarra, Alissa J. Rivera, David J. Arriola, Varykina G. Thackray

**Affiliations:** Department of Reproductive Medicine and the Center for Reproductive Science and Medicine, University of California San Diego, La Jolla, CA, United States of America; John Hopkins University School of Medicine, United States of America

## Abstract

In the present study, we investigate whether the FOXO1 transcription factor modulates activin signaling in pituitary gonadotropes. Our studies show that overexpression of constitutively active FOXO1 decreases activin induction of murine *Fshb* gene expression in immortalized LβT2 cells. We demonstrate that FOXO1 suppression of activin induction maps to the −304/−95 region of the *Fshb* promoter containing multiple activin response elements and that the suppression requires the FOXO1 DNA-binding domain (DBD). FOXO1 binds weakly to the −125/−91 region of the *Fshb* promoter in a gel-shift assay. Since this region of the promoter contains a composite SMAD/FOXL2 binding element necessary for activin induction of *Fshb* transcription, it is possible that FOXO1 DNA binding interferes with SMAD and/or FOXL2 function. In addition, our studies demonstrate that FOXO1 directly interacts with SMAD3/4 but not SMAD2 in a FOXO1 DBD-dependent manner. Moreover, we show that SMAD3/4 induction of *Fshb*-luc and activin induction of a multimerized SMAD-binding element-luc are suppressed by FOXO1 in a DBD-dependent manner. These results suggest that FOXO1 binding to the proximal *Fshb* promoter as well as FOXO1 interaction with SMAD3/4 proteins may result in decreased activin induction of *Fshb* in gonadotropes.

## Introduction

In mammalian reproduction, luteinizing hormone (LH) and follicle-stimulating hormone (FSH) production from pituitary gonadotrope cells is critical for the regulation of gonadal functions such as steroidogenesis and gametogenesis [1], [2]. LH and FSH are heterodimeric glycoproteins composed of a common alpha subunit and a beta subunit which is unique to each hormone [3]. Transcription of *Lhb* and *Fshb* is one of the rate limiting steps in the production of the mature hormones [4], [5] and is tightly controlled by a complex network of hormonal signaling pathways including those activated by gonadotropin-releasing hormone (GnRH) and activin [6].

Signals from pulsatile GnRH, released from the hypothalamus, are transmitted through activation of the G-protein coupled GnRH receptor on the surface of gonadotrope cells [7]. In addition to GnRH, activin signaling via binding to activin type II serine/threonine kinase receptors, which results in the phosphorylation of activin type I receptors [8], is also important for gonadotropin production. Activation of these receptors results in the phosphorylation of downstream Sma- and mothers against decapentaplegic (MAD)-related proteins, SMAD2 and SMAD3 [9]–[11]. SMAD2/3 then bind to SMAD4, translocate into the nucleus and activate transcription of specific target genes [8], [12], [13]. Activin responsiveness of the rodent *Fshb* promoter has been extensively characterized (reviewed in [14], [15]). SMAD2/3/4 have been shown to bind three SMAD binding elements (SBE) at −267, −149 and −116 of the murine *Fshb* promoter [11], [16]–[19]. Forkhead box L2 (FOXL2) has also been reported to bind three elements at −350, −154 and −113 in the murine *Fshb* promoter and mutation of these sites disrupt activin induction [18]–[21].

There is considerable evidence that gonadotropin production may be modulated by metabolic hormones such as insulin, in addition to reproductive hormones [22]–[27]. One group of candidate genes that may be regulated by insulin in gonadotropes is the FOXO subfamily of forkhead box transcription factors. FOXOs have been shown to be key regulators of cellular pathways involved in apoptosis, stress resistance, cell cycle arrest, and DNA damage repair [28], [29]. They also have important roles in metabolism, homeostasis and reproduction. *Foxo3* knockout mice have an age-dependent reduction in fertility caused by defective ovarian follicular growth, similar to premature ovarian failure in women [30]. Conditional knockouts of *Foxo1* have demonstrated that FOXO1 plays a role in ovarian granulosa cell proliferation and apoptosis, along with FOXO3 and that FOXO1 is essential for maintenance and differentiation of spermatogonial stem cells in the testis [31], [32]. The activity of FOXOs is regulated by post-translational modifications including phosphorylation, acetylation and ubiquitination [33]. Activation of the PI3K/AKT signaling pathway, in response to insulin/growth factor stimulation, results in FOXO phosphorylation, nuclear export and inhibition of their transcriptional activities [34].

Previously, we reported that the FOXO1 transcription factor is expressed in gonadotrope cells and that its phosphorylation and cellular localization are regulated by insulin signaling in a PI3K-dependent manner [35]. We also demonstrated that FOXO1 overexpression inhibits basal and GnRH induction of *Lhb* and *Fshb* synthesis in immortalized gonadotrope cells [35], [36]. Since FOXO1 was reported to interact with SMAD3/4 in immortalized keratinocytes [37], we hypothesized that FOXO1 may also modulate activin signaling in gonadotrope cells. In this study, we used the immortalized gonadotrope-derived LβT2 cell model to determine whether FOXO1 alters activin induction of *Fshb* gene expression and to investigate the mechanisms involved.

## Materials and Methods

### Plasmid Constructs

The pcDNA3 human FOXO1 and FOXO1-CA expression plasmids were previously described [38]. The pALTER human FOXO1, FOXO1-CA, and FOXO1-CA-DNA binding domain (DBD) mutant (W209G/H215L) expression vectors were generously provided by Dr. Terry Unterman [39]. The pRK5 SMAD2, SMAD3 and SMAD4 expression vectors were kindly provided by Dr. Rik Derynck. The −1000 murine *Fshb*-luciferase (luc) in pGL3 and 5′ truncations (−500, −304, −95) were previously described [18], [40], [41]. The 4XSBE-luc containing four repeats of a consensus SBE (GATCAGATCTGA) was obtained from Dr. Djurdjica Coss. The 4×FBE-luc was constructed by inserting four repeats of a consensus Forkhead binding element (FBE) (CCGTAAACAACT) upstream of a minimal thymidine kinase promoter in pGL3 using KpnI and NheI restriction enzyme sites as was the 4XFL2BE-luc containing four repeats of a consensus FOXL2 binding element (FLRE) (CCGTCAAGGTCT) [42].

### Tissue Cell Culture

Cell culture was performed with the immortalized murine LβT2 cell line which has many characteristics of a mature, differentiated gonadotrope [43], [44]. Cells were maintained in 10 cm plates in Dulbecco's Modification of Eagles Medium (DMEM) from Mediatech Inc., (Herndon, VA) with 10% Fetal Bovine Serum (FBS) (Omega Scientific, Inc., Tarzana, CA) and penicillin/streptomycin antibiotics (Gibco/Invitrogen, Grand Island, NY) at 37°C and 5% CO_2_. 1× Trypsin-EDTA (Sigma-Aldrich, St. Louis, MO) was used for cell dissociation.

### Transient Transfection

LβT2 cells were seeded at 4.5×10^5^ cells/well on 12-well plates and transfected 18 hours later, using PolyJet DNA *In Vitro* Transfection Reagent (SignaGen, Rockville, MD), following the manufacturer's instructions. For all experiments, the cells were transfected for 6 hours with 400 ng of the indicated luc reporter plasmid and 200 ng of a β-galactosidase (β-gal) reporter plasmid driven by the Herpes Virus thymidine kinase promoter to control for transfection efficiency. The cells were switched to serum-free DMEM containing 0.1% BSA, 5 mg/L transferrin and 50 mM sodium selenite 6 hours after transfection. After overnight incubation in serum-free media, the cells were treated with vehicle (0.1% BSA) or 10 ng/mL activin (Calbiochem, La Jolla, CA) for 6 hours.

### Luciferase and β-galactosidase Assays

To harvest the cells, they were washed with 1× phosphate buffered saline (PBS) and lysed with 0.1 M K-phosphate buffer pH 7.8 containing 0.2% Triton X-100. Lysed cells were assayed for luc activity using a buffer containing 100 mM Tris-HCl pH 7.8, 15 mM MgSO_4_, 10 mM ATP, and 65 µM luciferin. β-Gal activity was assayed using the Tropix Galacto-light assay (Applied Biosystems, Foster City, CA), according to the manufacturer's protocol. Both assays were measured using a Veritas Microplate Luminometer (Promega, Madison, WI).

### Statistical Analyses

Transient transfections were performed in triplicate and each experiment was repeated at least three times as indicated in the figure legend. The data were normalized for transfection efficiency by expressing luc activity relative to β-gal and then made relative to the empty pGL3 plasmid to control for FOXO1 effects on the empty vector. The data were analyzed by Student's *t*-test for independent samples, one-way analysis of variance (ANOVA) followed by post-hoc comparisons with the Tukey-Kramer Honestly Significant Difference test or two-way ANOVA to determine synergy as described in [45] using the statistical package JMP 11.0 (SAS, Cary, NC). Significant differences were designated as *p*<0.05.

### Adenoviral Infection

Adenoviral vectors containing cDNA of green fluorescent protein (Ad-GFP) and constitutively active FOXO1 (T24A/S256D/S319A) (Ad-FOXO1-CA) were provided by Dr. Domenico Accili [46]. LβT2 cells were seeded at 2×10^6^ cells/well on 6-well plates. The next morning, cells were transduced with a multiplicity of infection of 200 of Ad-GFP or Ad-FOXO1-CA for 6 hours, then switched to serum-free media. 24 hours after adenoviral infection, cells were treated with vehicle (0.1% BSA), 10 ng/mL activin, 10 nM GnRH (Sigma-Aldrich), or both hormones for 6 hours.

### Quantitative RT-PCR

Total RNA was extracted from LβT2 cells with TRIzol Reagent (Life Technologies, Carlsbad, CA) following the manufacturer's protocol. Contaminating DNA was removed with DNA-*free* reagent (Life Technologies). 2 µg of RNA was reverse-transcribed using the iScript cDNA Synthesis Kit (Bio-Rad Laboratories, Inc., Hercules, CA) according to the manufacturer's protocol. Quantitative real-time PCR was performed in an iQ5 iCycler using iQ SYBR Green Supermix (Bio-Rad Laboratories, Inc.) and the following primers: *Fshb* forward, GCCGTTTCTGCATAAGC; *Fshb* reverse, CAATCTTACGGTCTCGTATACC; Gapdh forward, TGCACCACCAACTGCTTAG; Gapdh reverse, GGATGCAGGGATGATGTTC, under the following conditions: 95°C for 5 min, followed by 40 cycles at 95°C for 45 sec, 54°C for 45 sec, and 72°C for 45 sec. Each sample was assayed in triplicate and the experiment was repeated three times. Standard curves with dilutions of a plasmid containing *Fshb* or *Gapdh* cDNA were generated with the samples in each run. In each experiment, the amount of *Fshb* was calculated by comparing the threshold cycle obtained for each sample with the standard curve generated in the same run. Replicates were averaged and divided by the mean value of *Gapdh* in the same sample. After each run, a melting curve analysis was performed to confirm that a single amplicon was generated in each reaction.

### Western Blot Analysis

Cells were harvested by incubating in a lysis buffer [10 mM Tris-HCl, pH 7.4, 150 mM NaCl, 1% Nonidet P-40 (NP40), 1 mM EDTA, 1 mM phenylmethylsulfonyl fluoride, complete protease inhibitor cocktail pellet (Roche Molecular Biochemical, Indianapolis, IN) and phosphatase inhibitor cocktail pellet (Roche)] for 10 min at 4°C. The protein concentration was determined by Bradford assay. An equal amount of protein per sample was loaded on a 10% SDS-PAGE gel. Proteins were resolved by electrophoresis and transferred for 2 h at 100 V onto polyvinylidene difluoride membrane (Millipore, Billerica, MA). Membranes were blocked overnight in 5% nonfat milk, then incubated overnight at 4°C with rabbit anti-human FOXO1 (1∶1000; sc-11350) or rabbit anti-human GAPDH (1∶3000; sc-25778). Blots were then incubated with an anti-rabbit horseradish peroxidase-linked secondary antibody (Santa Cruz Biotechnology) and bands were visualized using the SuperSignal West Dura Substrate (Thermo Scientific, Rockford, IL).

### Electrophoretic Mobility Shift Assay (EMSA)

Flag-FOXO1-CA was transcribed and translated using a TnT Coupled Reticulolysate System (Promega). The oligonucleotides were end-labeled with T4 polynucleotide kinase and [γ-^32^P] ATP. 4 µL of TnT lysate was incubated with 1 fmol of ^32^P-labeled oligo at 4°C for 30 min in a DNA-binding buffer [10 mM Hepes pH 7.8, 50 mM KCl, 5 mM MgCl_2_, 0.1% NP-40, 1 mM dithiothreitol, 2 µg poly(dI-dC), and 10% glycerol]. After 30 min, the DNA binding reactions were run on a 5% polyacrylamide gel (30:1 acrylamide: bisacrylamide) containing 2.5% glycerol in a 0.25× TBE buffer. Murine Flag M2 (Sigma-Aldrich F1804) antibody was used for supershift; murine IgG was used as a control for non-specific binding. The following oligonucleotides were used for EMSA: −305/−271 5′- GGATTCTGAGTTCGCCAAGTTAAAGATCAGAAAGA-3′, −275/−241 5′- AAAGAATAGTCTAGACTCTAGAGTCACATTTAATT-3′, −245/−211 5′- TAATTTACAAGGTGAGGGAGTGGGTGTGCTGCCAT-3′, −215/−181 5′- GCCATATCAGATTCGGTTTGTACAGAAACCATCAT-3′, −185/−151 5′- ATCATCACTGATAGCATTTTCTGCTCTGTGGCATT-3′, −155/−121 5′ GCATTTAGACTGCTTTGGCGAGGCTTGATCTCCCT-3′, −125/−91 5′- TCCCTGTCCGTCTAAACAATGATTCCCTTTCAGCA-3′, and the consensus FBE [47] 5′-CTAGATGGTAAACAACTGTGACTAGTAGAACACGG-3′.

### GST Interaction Assay

GST-SMAD2/3/4 were provided by Dr. Rik Derynck and the GFP expression vector by Dr. Douglass Forbes. ^35^S-labeled proteins were produced using the TnT Coupled Reticulolysate System. Bacteria transformed with GST plasmids were grown to OD of 0.6 and induced with IPTG overnight at 30°C [48]. Bacterial pellets were sonicated in 0.1% Triton X-100, 5 mM EDTA in 1× PBS, centrifuged and the supernatant was bound to glutathione sepharose 4B resin (Amersham Pharmacia Biotech, Piscataway, NJ). Beads were washed 4× in PBS and in HND buffer (10 mg/ml BSA, 20 mM Hepes pH 7.8, 50 mM NaCl, 5 mM DTT, and 0.1% NP-40). For the interaction assay, 20 µl of ^35^S-labeled *in vitro* transcribed and translated GFP, FOXO1, FOXO1-CA, or FOXO1-CA-DBD mutant was added to the beads with 400 µl of HND buffer. Beads were incubated overnight at 4°C, washed 2× with HND buffer and 2× with 0.1% NP-40 in PBS. Thirty µl of 2× Laemmli load buffer was added, the samples were boiled and electrophoresed on a 10% SDS-polyacrylamide gel. One fourth of the ^35^S-labeled *in vitro* transcribed-translated product was loaded onto the gel as input.

### Co-Immunoprecipitation Assay

LβT2 cells were incubated overnight in serum-free media and then treated with or without 10 ng/mL activin for 2 hours. The cells were harvested and nuclear extracts were prepared, as previously described [49]. Protein concentration was determined by Bradford assay. 400 µg of pre-cleared nuclear extracts were incubated with 4 µg of mouse IgG (Santa Cruz sc-2025), SMAD4 antibody (sc-7966) or SMAD2/3 antibody (BD Biosciences 610842) at 4°C for 1 hour. Twenty-five µL of Protein A Magnetic Beads (New England Biolabs, Ipswich, MA) were added and the extracts were rocked overnight at 4°C. Bead/protein complexes were washed 1× with PBS then eluted in 2× SDS sample buffer at 70°C for 5 minutes. 20 µg of protein was electrophoresed on a 10% SDS-PAGE gel, transferred to a polyvinylidene difluoride membrane and blocked overnight in 5% non-fat dry milk in 1× Tris-buffered saline with 0.1% Tween-20. The blots were then incubated overnight at 4°C with rabbit anti-SMAD4 (Millipore 04-1033; 1∶1000 dilution), SMAD2/3 (sc-8332; 1∶1000) or FOXO1 (sc-11350, 1∶1000 dilution) primary antibodies. Blots were incubated with a goat anti-rabbit horseradish peroxidase-linked secondary antibody (Santa Cruz; 1∶5000) and bands were visualized using the SuperSignal West Dura Substrate (Thermo Scientific).

## Results

### Constitutively Active FOXO1 Decreases Activin-Induced *Fshb*-luc

**Figure 1:**
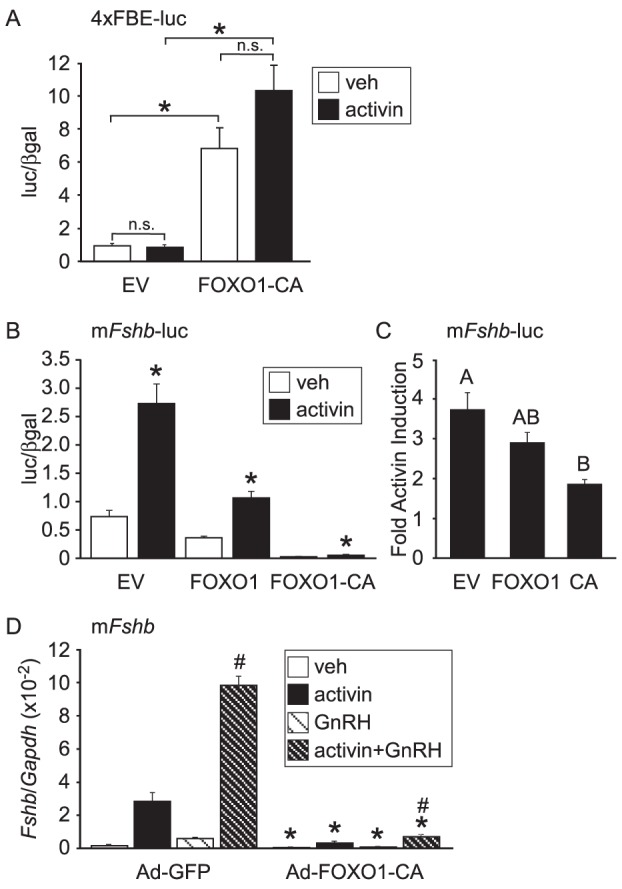
Overexpression of Constitutively Active FOXO1 Reduces Activin Induction of *Fshb* Transcription in LβT2 Cells. A. The 4×FBE-luc plasmid was transiently transfected into LβT2 cells along with 200 ng of pcDNA3 empty vector (EV) or FOXO1-CA expression vector, as indicated. After overnight incubation in serum-free media, cells were treated for 6 h with 0.1% BSA vehicle (veh) or 10 ng/mL activin. The results represent the mean ± SEM of three experiments performed in triplicate and are presented as luc/β-gal. * indicates that the induction by FOXO1-CA is significantly different from EV using Student's *t*-test while n.s. indicates that the activin induction is not significantly different from vehicle. B–C. The −1000 murine *Fshb*-luc plasmid was transfected into LβT2 cells along with pcDNA3 EV, FOXO1 or FOXO1-CA (CA), as indicated. After overnight incubation in serum-free media, cells were treated for 6 h with 0.1% BSA veh or 10 ng/mL activin. The results represent the mean ± SEM of three experiments performed in triplicate and are presented as luc/βgal (B) or fold activin induction relative to vehicle control (C). * indicates that there is a significant activin induction compared to vehicle using Student's *t*-test (B). The different uppercase letters indicate that fold activin induction is significantly repressed by FOXO1-CA compared to EV using one-way ANOVA followed by Tukey's post-hoc test (C). D. LβT2 cells were transduced with a multiplicity of infection of 200 of Ad-GFP or Ad-FOXO1-CA for 6 hours, then switched to serum-free media. 24 hours after adenoviral infection, cells were treated with 0.1% BSA veh, 10 ng/mL activin, 10 nM GnRH, or both hormones for 6 hours, as indicated. The results represent the mean ± SEM of three experiments performed in triplicate and are presented as amount of *Fshb* mRNA relative to *Gapdh*. * indicates that *Fshb* transcription is significantly repressed by FOXO1-CA compared to Ad-GFP using Student's *t*-test while # indicates synergy between activin and GnRH activin using two-way ANOVA. The data concerning the effect of veh vs. GnRH was published previously [36].

We recently published that overexpression of the FOXO1 transcription factor in immortalized LβT2 gonadotrope cells resulted in decreased basal and GnRH-induced *Lhb* and *Fshb* gene expression [35], [36]. To determine whether FOXO1 can modulate activin signaling in gonadotropes, we transfected LβT2 cells with a multimer containing four repeats of a consensus FBE fused with a luc reporter gene (4×FBE-luc) along with constitutively active FOXO1 (FOXO1-CA), which remains in the nucleus due to the inability of insulin/growth factor signaling to phosphorylate the mutated residues. Overexpression of FOXO1-CA increased expression of the 4×FBE-luc but activin treatment did not result in significantly increased transcription of the 4×FBE-luc in the absence or presence of FOXO1-CA (Fig. 1A). In contrast to the 4×FBE-luc, overexpression of FOXO1 reduced expression of −1000 bp of the murine *Fshb* promoter fused to a luc reporter gene (m*Fshb*-luc). As previously reported [36], both wild-type FOXO1 and FOXO1-CA reduced basal expression of m*Fshb*-luc (Fig. 1B). Additionally, although the fold activin induction of the murine *Fshb* promoter was not significantly decreased by wild-type FOXO1, FOXO1-CA significantly reduced activin induction of *Fshb* by 50% (Fig. 1C). The lack of a significant decrease in activin induction of *Fshb* due to overexpression of wild-type FOXO1 was not altogether unexpected since we previously showed that transfection of LβT2 cells with pcDNA3 FOXO1 resulted in FOXO1 being predominantly localized in the cytoplasm with some nuclear localization whereas pcDNA FOXO1-CA was localized in the nucleus [36].

### FOXO1 Decreases Activin-Induced *Fshb* mRNA Levels

To determine whether FOXO1-CA suppression of activin-induced transcription also occurs on the endogenous *Fshb* promoter in gonadotropes, we transfected LβT2 cells with a control GFP adenovirus (Ad-GFP) or an adenovirus containing FOXO1-CA (Ad-FOXO1-CA) and measured *Fshb* mRNA levels relative to *Gapdh* in response to vehicle, activin, GnRH, or activin and GnRH co-treatment. As previously reported [36], overexpression of FOXO1-CA significantly decreased basal *Fshb* mRNA levels by 62% (Fig. 1D). In LβT2 cells transduced with Ad-GFP, *Fshb* mRNA levels were induced 17 fold by activin and 4 fold by GnRH while cotreatment with activin and GnRH resulted in a synergistic 60 fold induction, similar to what was previously reported [50], [51]. In contrast, activin induction was decreased by 62%, GnRH by 65% and activin and GnRH synergy by 82% in cells transduced with Ad-FOXO1-CA (Fig. 1D). These results indicate that constitutively active FOXO1 suppression of activin-, GnRH- or activin and GnRH-induced *Fshb* gene expression occurs in the context of the native chromatin.

### FOXO1 Repression of Activin Induction Maps Between −304 and −95 of the *Fshb* Promoter

**Figure 2:**
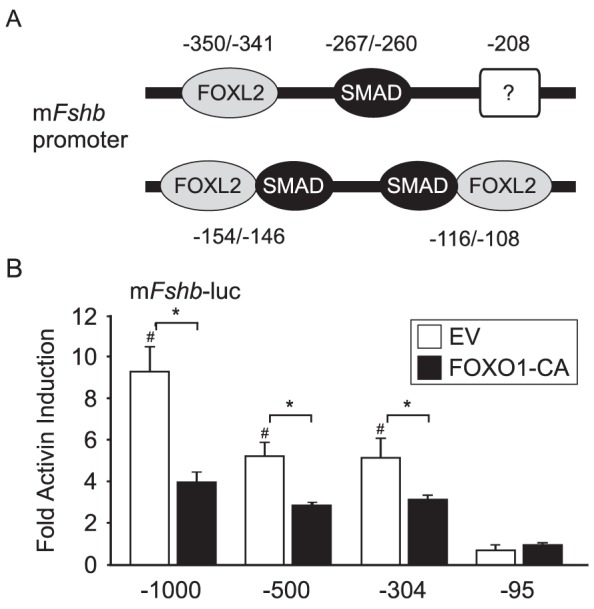
FOXO1 Suppression Maps to −304/−95 of the Murine *Fshb* Promoter. A. Diagram illustrating the location of activin response elements on the murine *Fshb* promoter including SMAD and FOXL2 binding sites. B. The −1000, −500, −304, and −95 murine *Fshb*-luc plasmids were transiently transfected into LβT2 cells along with pcDNA3 empty vector (EV) or FOXO1-CA, as indicated. After overnight incubation in serum-free media, cells were treated for 6 h with 0.1% BSA or 10 ng/mL activin. The results represent the mean ± SEM of three experiments performed in triplicate and are presented as fold activin induction relative to the vehicle control. # indicates that *Fshb* transcription is significantly induced by activin compared to vehicle using Student's *t*-test while * indicates that fold activin induction is significantly repressed by FOXO1-CA compared to EV using Student's *t*-test.

As described in the introduction, the murine *Fshb* promoter contains multiple activin response elements including SMAD and FOXL2 binding sites (Fig. 2A). We used 5′ truncation analysis to determine which regions of the promoter were necessary for FOXO1 suppression. Both activin induction and FOXO1 suppression were lost with the −95 *Fshb*-luc (Fig. 2B). These results indicate that the region between −304 and −95 is necessary for activin responsiveness as well as suppression by FOXO1 and suggest that the mechanism of suppression may involve SMAD and FOXL2 transcription factors.

### FOXO1 DBD Is Required for Suppression of *Fshb* Gene Expression

**Figure 3:**
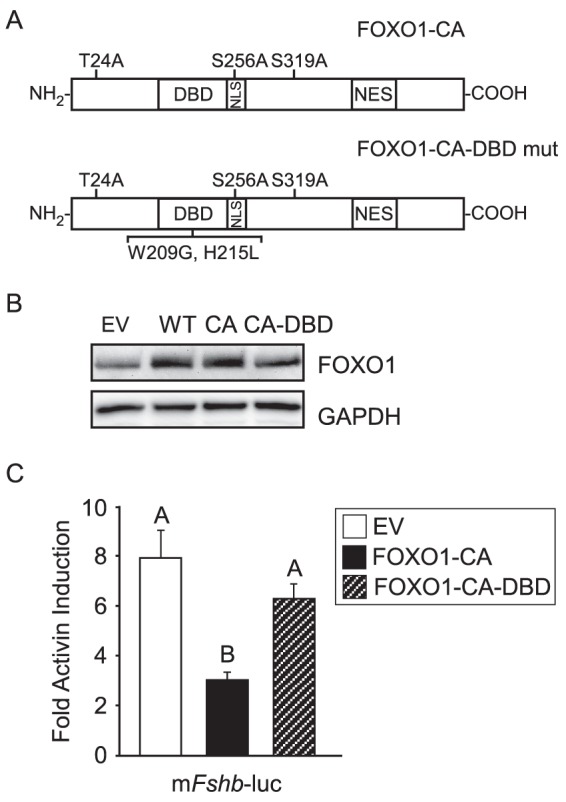
FOXO1 DNA Binding Domain Is Required to Suppress Activin-Induced *Fshb* Gene Expression. A. Diagram illustrating FOXO1-CA-DBD mutant (W209G/H215L). (B) LβT2 cells were transfected with pALTER empty vector (EV), FOXO1 (WT), FOXO1-CA (CA), or FOXO1-CA-DBD (CA-DBD) for 6 hours, then switched to serum-free media. Twenty-four hours after transfection, the cells were harvested. Western blot analysis was performed on whole cell extracts using FOXO1 and GAPDH primary antibodies and a horseradish peroxidase–linked secondary antibody. A representative image is shown. C. The −1000 murine *Fshb*-luc reporter was transfected into LβT2 cells along with pALTER EV, FOXO1-CA or FOXO1-CA-DBD mutant, as indicated. After overnight incubation in serum-free media, cells were treated for 6 h with 0.1% BSA or 10 ng/mL activin. The results represent the mean ± SEM of three experiments performed in triplicate and are presented as fold activin induction relative to the vehicle control. The different uppercase letters indicate that fold activin induction is significantly repressed by FOXO1-CA compared to EV or FOXO1-CA-DBD using one-way ANOVA followed by Tukey's post-hoc test.

To further investigate how activin-induced *Fshb* transcription is inhibited by FOXO1, we tested whether the FOXO1 DBD was necessary for the repression, as previously demonstrated for FOXO1 suppression of basal and GnRH-induced *Lhb* and *Fshb* gene expression [35], [36]. As a control for the level of protein expression, we demonstrated that comparable levels of FOXO1-CA and a FOXO1-CA-DBD mutant were expressed when transfected into LβT2 cells (Fig. 3B). While FOXO1-CA overexpression in LβT2 cells suppressed activin-induced *Fshb*-luc, overexpression of FOXO1-CA with a DBD mutation (FOXO1-CA-DBD, Fig. 3A) was not able to repress activin induction of *Fshb* (Fig. 3C). These results indicate that the FOXO1 DBD is necessary to elicit an inhibitory effect on activin signaling to the *Fshb* promoter.

### FOXO1 Binds to the Proximal *Fshb* Promoter

**Figure 4:**
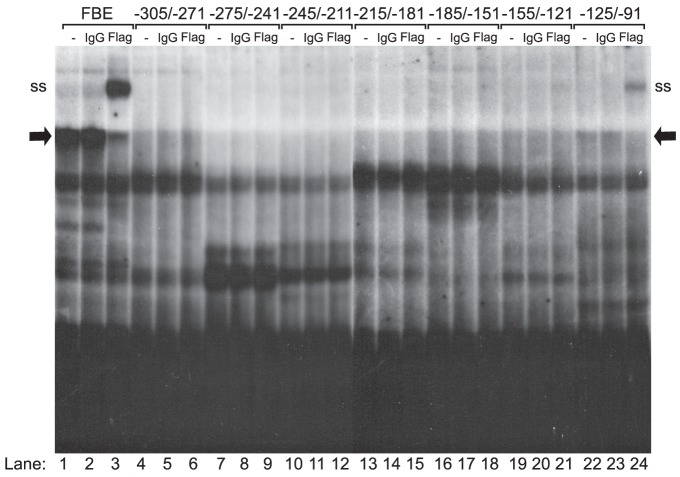
FOXO1 Binds to −125/−91 of the *Fshb* Promoter. TnT Flag-FOXO1-CA was incubated with a consensus FBE, −305/−271, −275/−241, −245/−211, −215/−181, −185/−151, −155/−121, or −125/−91 *Fshb* probes and tested for complex formation in EMSA. FOXO1-CA-DNA complex on the FBE is shown in lane 1, IgG control in lane 2 and Flag supershift is shown in lane 3. The FOXO1-CA-DNA complex (arrow) and antibody supershift (ss) are indicated on the left and right of the gel.

Since the FOXO1 repression mapped to the −304/−95 region of the *Fshb* promoter and required the FOXO1 DBD, we performed EMSA to determine whether FOXO1 could bind to this part of the promoter *in vitro*. Seven 35-mer oligonucleotide probes were designed to span the −304/−95 region. Flag-FOXO1-CA, synthesized with TnT rabbit reticulocyte lysate, bound to an oligonucleotide probe containing a consensus FBE (Fig. 4A, lane 1). To identify which complex contained the Flag-FOXO1-CA bound to the FBE, we supershifted the complex with a Flag antibody (Fig. 4A, lane 3) but not with control IgG (Fig. 4A, lane 2). Incubation with an oligo encompassing the −125/−91 region of the *Fshb* promoter also resulted in the formation of a barely detectable protein-DNA complex that was clearly shifted with a Flag antibody but not IgG (Fig. 4A, lanes 22–24) while incubation with oligos encompassing the −305/−121 regions did not result in detectable FOXO1 binding (Fig. 4A, lanes 4–21). These results suggest that, in contrast to the consensus FBE, FOXO1 can bind weakly to the −125/−91 region of the murine *Fshb* promoter.

### FOXO1 Interacts with SMAD3 and SMAD4

**Figure 5:**
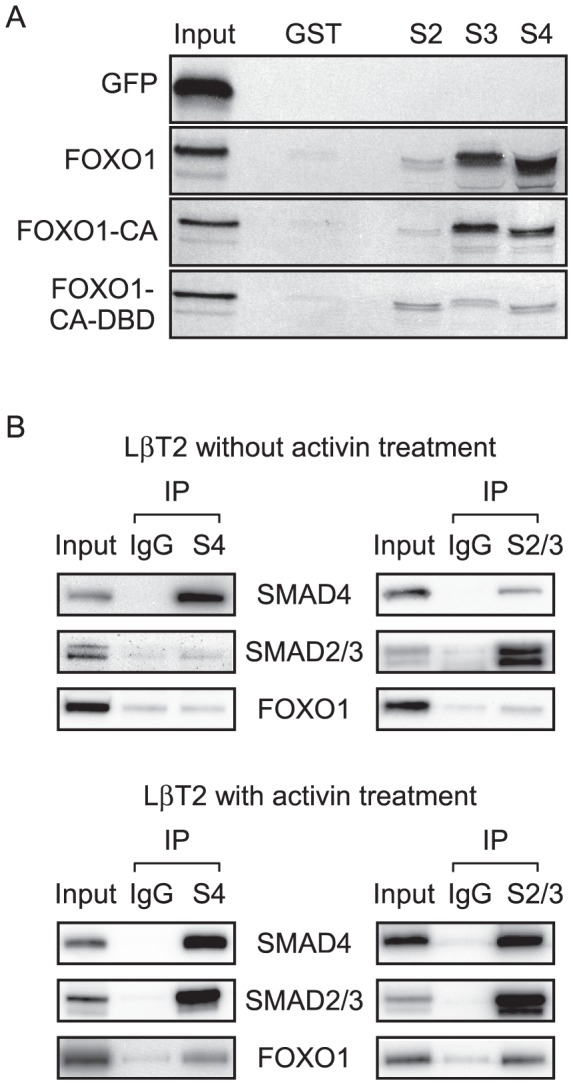
FOXO1 Interacts with SMAD3/4. A. GST interaction assays were performed using bacterially expressed GST-fusion proteins (indicated above each lane) and ^35^S-labeled *in vitro* transcribed and translated GFP, FOXO1, FOXO1-CA, and FOXO1-CA-DBD mutant (indicated on the left of the panel). GFP was used as a negative control. The GST-fusion proteins included GST alone, GST-SMAD2 (S2), GST-SMAD3 (S3), and GST-SMAD4 (S4). One quarter of the protein used in the interaction assay was loaded in the lane marked input. The experiment was repeated several times with similar results and a representative experiment is shown. B. Co-immunoprecipitation assays were performed using nuclear extracts from LβT2 cells treated with or without 10 ng/mL activin for 2 h after an overnight incubation in serum-free media. One tenth of the protein used in the immunoprecipitation reaction was loaded in the lane marked input. The immunoprecipitation was performed with mouse IgG, SMAD4 (S4) or SMAD2/3 (S2/3) antibody, as indicated. Western blot analysis was performed using SMAD4, SMAD2/3 and FOXO1 primary antibodies, and a horseradish peroxidase-linked secondary antibody. The experiment was repeated several times with similar results and a representative experiment is shown.

Since FOXO1 binding to the −125/−95 region of the *Fshb* promoter was weak compared to FOXO1 binding to the consensus FBE, we investigated whether FOXO1 physically interacts with SMAD proteins. We tested whether FOXO1 or DNA-binding deficient FOXO1 interacts with SMADs by incubating GST-SMAD2/3/4 fusion proteins with *in vitro*-transcribed and translated ^35^S-labeled FOXO1, FOXO1-CA or a FOXO1-CA-DBD mutant in pull-down experiments. As shown in Fig. 5A, there was minimal interaction between the GST-SMAD fusion proteins and the negative control (^35^S-GFP) or with GST alone incubated with FOXO1, FOXO1-CA or the FOXO1-CA-DBD mutant. In contrast, there was a strong interaction between FOXO1 and SMAD3 or SMAD4 which was not observed between FOXO1 and SMAD2. Additionally, a strong interaction was also observed between FOXO1-CA and SMAD3 or SMAD4 while there was no detectable interaction between the FOXO1-CA-DBD mutant and SMAD3 or SMAD4, indicating that the interaction between FOXO1 and SMAD3/4 requires the FOXO1 DBD.

Given our results demonstrating a direct protein-protein interaction between FOXO1 and SMAD3/4 *in vitro*, we then determined whether endogenous FOXO1 could interact with these proteins in gonadotrope cells using a co-immunoprecipitation assay. As shown in Fig. 5B, SMAD4 and SMAD2/3 were efficiently immunoprecipitated from nuclear extracts obtained from activin-treated or untreated LβT2 cells. Since SMAD4 has been previously shown to interact with SMAD2/3 [52], SMAD2/3 co-immunoprecipitated with SMAD4 or *vice versa* were used as positive controls and occurred in cells treated with activin. Interestingly, FOXO1 was also co-immunoprecipitated with SMAD4 or SMAD2/3 in an activin-dependent manner, indicating that endogenous FOXO1 can interact with SMAD3 and SMAD4 in gonadotropes.

### FOXO1 Suppression of Activin-Induced *Fshb* Transcription Involves Inhibition of SMAD Transcription Factors

**Figure 6:**
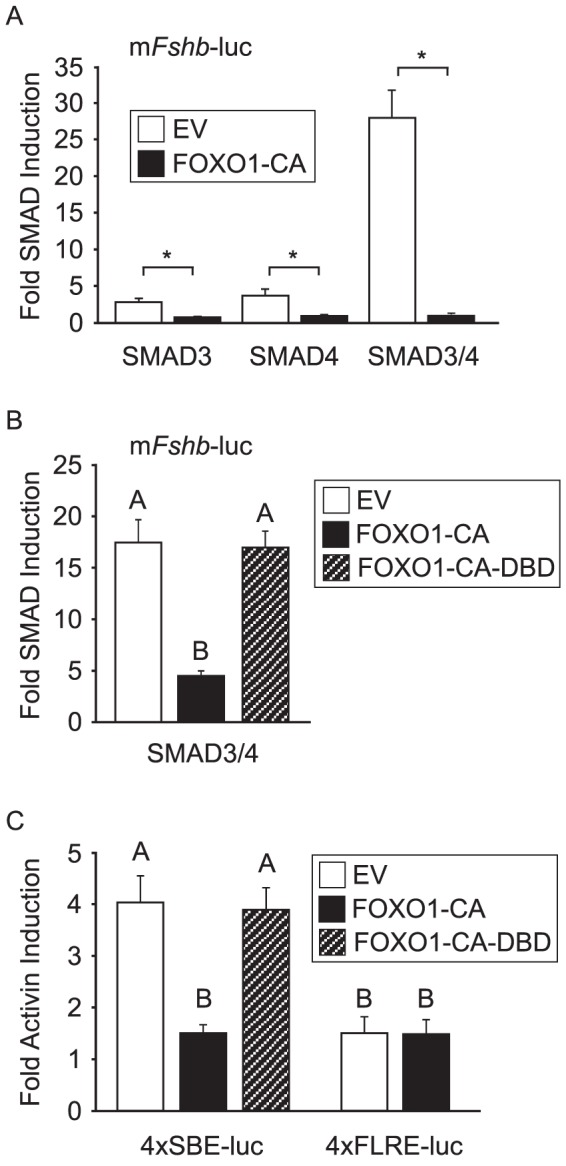
FOXO1 Suppresses SMAD-Induced *Fshb* Gene Expression. A–B. The −1000 murine *Fshb*-luc plasmid was transfected into LβT2 cells along with 100 ng of pALTER empty vector (EV), FOXO1-CA or FOXO1-CA-DBD mutant, as well as 100 ng of pRK5 EV, 50 ng SMAD3 or SMAD4 with 50 ng of pRK5, or 50 ng SMAD3 and SMAD4 expression vectors, as indicated. Cells were incubated in serum-free media for 24 h before harvest. The results represent the mean ± SEM of three experiments performed in triplicate and are presented as fold SMAD induction relative to the pRK5 EV. * indicates that *Fshb* transcription is significantly repressed by FOXO1-CA compared to EV using Student's *t*-test (A) while the different uppercase letters indicate that *Fshb* transcription is significantly repressed by FOXO1-CA compared to EV or FOXO1-CA-DBD using one-way ANOVA followed by Tukey's post-hoc test (B). C. 4×SBE-luc or 4×FLRE-luc plasmids were transfected into LβT2 cells along with 200 ng of pALTER EV, FOXO1-CA or FOXO1-CA-DBD. After overnight incubation in serum-free media, cells were treated for 6 h with 0.1% BSA or 10 ng/mL activin. The results represent the mean ± SEM of three experiments performed in triplicate and are presented as fold activin induction relative to the vehicle control. The different uppercase letters indicate that transcription of the 4×SBE-luc is significantly reduced by FOXO1-CA compared to EV or FOXO1-CA-DBD using one-way ANOVA followed by Tukey's post-hoc test while there is no significant difference in transcription of the 4×FLRE-luc.

To further examine the mechanism of FOXO1 repression of activin induction, we tested whether FOXO1 could alter SMAD-dependent transcription in gonadotrope cells. Initially, we examined the effect of FOXO1-CA overexpression on SMAD induction of *Fshb* gene expression. Overexpression of SMAD3 or SMAD4 induced *Fshb* transcription by 3–4 fold while overexpression of SMAD3 and SMAD4 resulted in a 28-fold induction (Fig. 6A). Noticeably, FOXO1-CA overexpression resulted in a profound inhibition of SMAD3/4 induction of *Fshb* synthesis (Fig. 6A). We also demonstrated that the FOXO1 DBD was required for the suppression of SMAD3/4-induced *Fshb* transcription (Fig. 6B). Since FOXO1 suppression of activin-induced *Fshb* transcription mapped to the −304/−95 region of the *Fshb* promoter that contains multiple SMADs and FOXL2 binding sites, we tested whether activin induction of a multimer containing four repeats of a consensus binding element for SMADs or FOXL2 (SBE or FLRE) was inhibited by FOXO1. Activin induced the 4×SBE-luc by 4 fold while the 4×FLRE-luc was induced by 1.5 fold (Fig. 6C). Interestingly, overexpression of FOXO1-CA significantly reduced the fold activin induction of the SBE but had no effect on the FLRE (Fig. 6C). Additionally, overexpression of FOXO1-CA with a DBD mutation was unable to suppress activin induction of the SBE, indicating that the FOXO1 DBD was also required for this effect. The weak activin induction of the 4×FLRE and the lack of activin induction of a multimer of the −350/−341 FOXL2 binding site [19] suggest that, unlike an SBE, a FOXL2 binding site is not sufficient for activin induction and thus, it is difficult to assess the role of FOXL2 in the FOXO1 suppression. In contrast, our data provides strong evidence that overexpression of FOXO1-CA results in decreased activin and SMAD-induction of *Fshb* in immortalized gonadotrope cells.

## Discussion

The importance of activin signaling in the production of FSH has been illustrated using mouse knockout models. Deletion of the Type II activin receptor as well as gonadotrope-specific knockdown of both SMAD4 and FOXL2 resulted in a hypogonadal hypogonadism phenotype reminiscent of the *Fshb* knockout [53]–[55]. Since FOXO1 was previously reported to interact with SMAD3/4 proteins in HaCAT and Cos-1 cells [37], we investigated whether FOXO1 could regulate activin induction of *Fshb* gene expression. We demonstrated that overexpression of constitutively active FOXO1 repressed activin- or activin and GnRH-induced transcription of a murine *Fshb*-luc reporter as well as endogenous *Fshb* mRNA in LβT2 cells (Fig. 1). The FOXO1 repression occurred in a context-dependent manner since FOXO1 overexpression induced transcription of a consensus FBE (Fig. 1A). Along with evidence that FOXO1 can suppress basal and GnRH-induced *Fshb* gene expression [36], [56], these data support the idea that nuclear localized FOXO1 may have a significant inhibitory effect on *Fshb* transcription in gonadotrope cells although it should be noted that overexpression studies can result in responses that do not occur in the *in vivo* physiological context.

Since activin regulation of *Fshb* transcription involves both SMADs and FOXL2, we sought to characterize the mechanism that FOXO1 employs to repress activin signaling in gonadotropes. We demonstrated that the region between −304 and −95, which contains several SMAD and FOXL2 binding elements, is necessary for the FOXO1 repression (Fig. 2). We then showed that two mutations (W209G and H215L) in helix 3 of the FOXO1 DBD prevented FOXO1 from eliciting a repressive effect (Fig. 3). H215 has been shown to make DNA contacts through hydrogen-bonding and water-mediated interactions [57] but the role of these two residues in protein-protein interactions is unknown. Our data suggests that the FOXO1 DBD is necessary for FOXO1 repression because FOXO1 binds directly to the *Fshb* promoter or because FOXO1 forms protein-protein interactions with factors critical for activin induction of *Fshb* transcription via the FOXO1 DBD.

To assess these two possibilities, we tested whether FOXO1 could bind to the region of the *Fshb* promoter necessary for the repressive effect. Gel-shift assays demonstrated that *in vitro* transcribed and translated FOXO1 bound to the −125/−91 region of the *Fshb* promoter, albeit much more weakly than FOXO1 binding to a consensus FBE (Fig. 4). Interestingly, the −125/−91 region contains a composite binding element for SMAD and FOXL2 proteins which has been shown to be essential for activin induction [16], [20], [21]. We did not observe binding of FOXO1 to this region of the promoter when LβT2 nuclear extracts were used instead of TnT FOXO1. Our studies are in agreement with a recent report by Choi *et al.* which demonstrated that FOXO1 can bind to the *Fshb* promoter using a DNA pulldown assay but was not detectable in a gel-shift assay employing LβT2 nuclear extracts [56]. Thus, these studies indicate that FOXO1 binding to the *Fshb* promoter may be a potential mechanism of FOXO1 repression of activin signaling in gonadotropes.

We then explored the possibility that FOXO1 repression of activin signaling in gonadotropes is due to protein-protein interactions between FOXO1 and SMAD3/4. Our GST pulldown experiments confirmed that FOXO1 can directly interact with SMAD3 and SMAD4 *in vitro* and that this interaction was dependent on the FOXO1 DBD (Fig. 5A). Notably, this was in contrast to the lack of interaction observed between FOXO1 and SMAD2. Since SMAD2 and SMAD3 are very similar proteins except that SMAD2 contains an insertion in the MH1 domain that prevents DNA binding, our data suggests that the inhibitory domain prevents interaction between FOXO1 and SMAD2. This idea is supported by the fact that the SMAD2 splice variant that lacks this insertion was reported to bind FOXO proteins [37]. It is also noteworthy that SMAD3 and SMAD4 were previously reported to bind FOXO1 through the MH1 domain [37]. FOXO1 interaction with SMAD3 and SMAD4 in an activin-dependent manner in co-immunoprecipitation experiments suggests that SMAD phosphorylation is required to bind FOXO1 (Fig. 5B). This data is in agreement with the report that the FOXO1 interaction with SMAD3/4 was dependent on TGFβ treatment of the cells [37]. Our studies also demonstrated that FOXO1 repressed the effects of SMAD3/4 overexpression on *Fshb*-luc and activin induction of a 4×SBE-luc in a FOXO1 DBD-dependent manner (Fig. 6). Altogether, these results provide strong support for the hypothesis that FOXO1-CA modulates activin responsiveness of the *Fshb* promoter by interacting with SMAD3/4 via the FOXO1 DBD.

In summary, our studies provide evidence that the FOXO1 transcription factor may negatively regulate activin induction of *Fshb* synthesis through FOXO1 binding to the proximal *Fshb* promoter as well as through a direct interaction between the FOXO1 DBD and SMAD3/4. Since these experiments were performed in immortalized gonadotrope cells, additional studies are needed to determine whether FOXO1 functions in the pituitary to negatively regulate gonadotropin production *in vivo*. Moreover, since FOXO proteins act as coactivators of SMAD-dependent transcription in several other cell types [37], [58], [59], future studies are required to understand how FOXO1 acts as a repressor of activin signaling in pituitary gonadotrope cells. It may also be worthwhile to investigate whether FOXO1 regulates additional activin responsive genes in gonadotrope cells including the GnRH receptor and follistatin [60]–[62]. Interactions between FOXO and SMAD proteins may also be important for regulation of gene expression in other reproductive tissues that express both of these transcription factors such as the ovary and uterus [63], [64].
